# Inhibition of Melanin Synthesis and Inflammation by Exosomes Derived from *Leuconostoc mesenteroides* DB-14 Isolated from *Camellia japonica* Flower

**DOI:** 10.4014/jmb.2411.11080

**Published:** 2025-01-06

**Authors:** Byeong Min Choi, Tae-Jin Park, HuSang Harry Lee, Hyehyun Hong, Won-Jae Chi, Seung-Young Kim

**Affiliations:** 1 Department of Pharmaceutical Engineering & Biotechnology, Sunmoon University, Chungnam 31460, Republic of Korea; 2 Newton South High School, 02459 Massachusetts, USA; 3 Biodiversity Research Department, Species Diversity Research Division, Incheon 22689, Republic of Korea

**Keywords:** Anti inflammation, *Camella japonica*, *Leuconostoc mesenteroides* DB-14, MAPK, melanogenesis

## Abstract

*Leuconostoc mesenteroides* is a lactic acid bacteria found in fermented products. In our previous study, *L. mesenteroides* was isolated from *Camellia japonica* flowers, and its acid tolerance and antibacterial properties were thoroughly investigated. This study focuses on the inhibition of melanin synthesis and inflammation of exosomes derived from *L. mesenteroides*. Moreover, *L. mesenteroides* exosomes (DB-14 exosome) exhibited significant inhibitory effects on inflammation and melanogenesis. At concentrations of 4.44 × 10^8^, 8.88 × 10^8^, and 1.78 × 10^9^ particles/ml, the exosomes reduced nitric oxide and prostaglandin E2 activity while maintaining the growth of RAW 264.7 macrophages. In addition, proinflammatory cytokines, such as interleukin (IL)-1β, IL-6, and tumor necrosis factor alpha, were rarely expressed, and western blot revealed that *L. mesenteroides* DB-14 derived exosomes inhibited inducible nitric oxide synthase and cyclooxygenase-2 expression. Moreover, the exosomes had no toxic effects on B16F10 melanoma cells at concentrations of 1.78 × 10^9^, 3.55 × 10^9^, and 7.10 × 10^9^ particles/ml, and they suppressed melanogenesis by reducing tyrosinase activity. Furthermore, western blot analysis demonstrated that microphthalmia-associated transcription factor (MITF), tyrosinase, tyrosinase related protein (TRP)-1, and TRP-2 were evidently reduced, ultimately repressing melanin production. Moreover, MITF expression was inhibited by reduced mitogen-activated protein kinase and protein kinase B phosphorylation levels. Overall, this study proves the efficacy of the novel DB-14 exosome as a strong lightening and anti-inflammatory agent.

## Introduction

Lactic acid bacteria (LAB) are commonly divided into two main groups based on their habitat. One group includes animal-derived LAB, which inhabit the skin and digestive tracts of animals and are predominantly employed in dairy production. The other group consists of plant-derived LAB, widely utilized in the fermentation of foods such as kimchi. Depending on their source, LAB have been applied for various purposes, with plant-derived LAB, in particular, being reported to offer potential benefits such as alleviating constipation and enhancing liver function [1, 2].

Exosomes are extracellular vesicles that form and release in all cells and are considered a medium of intercellular communication [3-5]. Exosomes, characterized by their cell-like structure and particle size of 30–200 nm, are known for their high applicability in vivo and have been utilized as delivery vectors for pharmaceuticals [6-9]. The isolation and acquisition of exosomes are conducted using methods such as ultracentrifugation, exosome isolation kits based on sedimentation principles, and tangential flow filtration (TFF) systems. In this study, the TFF method was utilized [10, 11]. The ultracentrifugation method, while widely used, is associated with high costs and poses a risk of exosome damage due to the high pressures involved during the purification process. Similarly, exosome extraction kits, which operate based on precipitation principles, are limited in their ability to effectively separate impurities with larger particle sizes. Thus, using the TFF system to separate exosomes from media is essential when investigating their direct effects. This filtration system is largely favored in the biomedical field due to its ability to separate nanoparticles of a specific size from a sample [12, 13]. Ultimately, the TFF system provides a higher concentration of exosomes than that of the other filtration systems. Therefore, we used TFF to obtain pure exosomes from *L. mesenteroides* DB-14 culture media to test their effects on melanin synthesis and inflammation.

Among the various types of immune responses in the human body, inflammation acts against pathogens and injuries to tissue membranes [14, 15]. Melanogenesis is another common immune response [16]. Melanin is synthesized by melanocytes in the epidermis to protect skin cells from ultraviolet radiation and prevent complications, such as actinic keratosis and malignant melanoma [17, 18]. Although these two common immune responses often maintain homeostasis and have positive consequences, excessive chronic inflammation may cause cell apoptosis and genetic mutations, which can cause atopy, cardiovascular diseases, and even cancer [19-23]. Moreover, the excessive production of melanin molecules, also known as hypermelanogenesis, may cause cell apoptosis and melanoma due to the toxicity of precursor molecules [24, 25].

Inflammation is triggered when lipopolysaccharide (LPS), an endotoxin that activates macrophages, binds to toll-like receptor 4, activating the mitogen-activated protein kinase (MAPK) and nuclear factor-kappa B (NF-κB) pathways. These proinflammatory pathways allow macrophages to generate inducible nitric oxide synthase (iNOS) and cyclooxygenase 2 (COX-2), which stimulates the synthesis of nitric oxide (NO) and prostaglandin E2 (PGE_2_), respectively [26, 27]. Additionally, NO and PGE2 concurrently stimulate proinflammatory cytokines, such as tumor necrosis factor alpha (TNF-α), interleukin (IL)-6, and IL-1β, which are significantly associated with tumor induction and progression [28, 29]. In contrast, melanogenesis is stimulated when the skin is exposed to ultraviolet radiation from sunlight or under severe stress. Both are known to stimulate the central nervous system to release alpha melanocyte stimulating hormone (α-MSH) from the pituitary gland, which activate melanocytes. Melanocytes then induce the synthesis of microphthalmia-associated transcription factor (MITF), tyrosinase, tyrosinase-related protein (TRP)-1, and dopachrome tautomerase (TRP)-2 [30, 31]. This pathway leads to the production of eumelanin and pheomelanin, which may ultimately lead to hyperpigmentation and apoptosis. Therefore, it is crucial to control and regulate the synthesis of proinflammatory molecules and proteins closely associated with the early stages of melanogenesis. However, research exploring these issues with existing drugs or chemicals remains limited. To address this gap, this study focuses on utilizing novel symbiotic plant bacteria as potential treatment agents. This agent not only demonstrates unprecedented efficacy but also shows potential for clinical applications due to its high level of biocompatibility.

## Materials and Methods

### Incubation of *Leuconostoc mesenteroides* DB-14

The *Leuconostoc mesenteroides* DB-14 strain was isolated from *Camellia japonica* flowers collected on March 22, 2019, at Bulgap Mountain in Moak-ri, Bulgap-myeon, Yeonggwang-gun, Jeollanam-do, South Korea, and was provided by the National Institute of Biological Resources (NIBR, Republic of Korea). *L. mesenteroides* DB-14 was cultured under aerobic conditions at 30°C using de Man, Rogosa, and Sharpe (MRS) medium to support its growth.

### Exosome Isolation

After cultivating *L. mesenteroides* DB-14 at 30°C at 200 ×*g* for 18 h using MRS broth, the bacterial solution was centrifuged at 4,000 ×*g* for 15 min to obtain the supernatant. The supernatant solution was then filtered using a 0.22-μm decompression filter. Tangential flow filtration (TFF, Cytiva, USA) with a hollow fiber cartridge filter and a pore size of 100,000 NMWC was used to filter the exosomes from the solution.

### Characterization of *L. mesenteroides* DB-14-Derived Exosome

Nanoparticle tracking analysis (NTA) and transmission electron microscopy (TEM) were used to analyze the exosomes. NTA was performed using ZetaView PMX 110 (Particle Metrix, Germany) and ZetaView software (version 8.05.16 SP3). TEM imaging was performed using Alos L120C (FEI, SA) by loading exosome samples on the formvar-coated copper grid surface, staining with 2% uranyl acetate for 20 s, followed by blotting.

### Cell Culture

B16F10 melanoma and RAW 264.7 cells were obtained from the Korean Cell Line Bank. Dulbecco’s Modified Eagle’s Medium (DMEM, Welgene, Republic of Korea), which contained 10% fetal bovine serum, 100 μg/ml streptomycin, and 100 μg/ml penicillin, was used. Both cell lines were cultured in this medium at 37°C and 5% CO_2_. RAW 264.7 cells were subcultured every two days, whereas B16F10 cells were subcultured every three days. LPS and α-MSH, which were used as cell stimuli for numerous experiments, were obtained from Sigma-Aldrich.

### Measurement of Cell Viability

To analyze the effects of DB-14 exosome on α-MSH-induced B16F10 cells as well as LPS-induced RAW 264.7 cells, the 3-(4,5-dimethylthiazol-2-yl)-2,5-diphenyl-2H-tetrazolium bromide (MTT) assay was performed. First, B16F10 melanoma cells were seeded in 24-well plates at 1.0 × 10^4^ cells/well and preincubated at 37°C and 5% CO_2_ for 24 h. Subsequently, the cells were simultaneously treated with 200 nM of α-MSH and *L. mesenteroides* DB-14-derived exosome (DB-14 exosome, 4.44 × 10^8^, 8.88 × 10^8^, 1.78 × 10^9^ particles/ml) and incubated with the cells for 72 h. Similarly, RAW 264.7 cells were seeded in 24-well plates at 8.0 × 10^4^ cells/well and preincubated at 37°C and 5% CO_2_ for 24 h. The cells were then were simultaneously treated with LPS (1 μg/ml) and DB-14 exosome (1.78 × 10^9^, 3.55 × 10^9^, 7.10 × 10^9^ particles/ml) and incubated for 72 h. Thereafter, MTT reagent (Sigma-Aldrich) was added to each well and incubated in at 37°C and 5% CO_2_ for 3 h to form visible formazan blue. Subsequently, dimethyl sulfoxide (Sigma-Aldrich) was added to each well, the supernatant was removed to dissolve the formazan blue, and then transferred to a 96-well plate to measure the absorbance at 570 nm using a microplate reader (Thermo Fisher Scientific, USA).

### Nitric Oxide Production Measurements

To investigate the NO inhibitory activity of DB-14 exosome, RAW 264.7 cells were seeded into 24-well plates at 8.0 × 10^4^ cells/well and then incubated at 37°C and 5% CO_2_ for 24 h. Afterwards, the cells were simultaneously treated with LPS (1 μg/ml) and DB-14 exosome (4.44 × 10^8^, 8.88 × 10^8^, 1.78 × 10^9^ particles/ml). After 24 h, 100 μl of culture solution and Griess reagent, which contained 1% (w/v) sulfanilamide and 0.1% (w/v) naphthyl-ethylenediamine in 2.5% (v/v) phosphoric acid, were mixed in the same amount and subjected to dark reaction for 10 min. The absorbance was measured at 540 nm.

### PGE_2_ Production Measurements

To measure the inhibitory activity of DB-14 exosome against PGE_2_ expression, RAW 264.7 cells were seeded in 24-well plates at 8.0 × 10^4^ cells/well and preincubated in at 37°C and 5% CO_2_ for 24 h. Subsequently, the cells were simultaneously treated with LPS (1 μg/ml) and DB-14 exosome (4.44 × 10^8^, 8.88 × 10^8^, 1.78 × 10^9^ particles/ml) and reacted for 24 h. The culture medium was recovered and centrifuged at 10,000 ×*g* for 3 min and the precipitate was removed. The remaining supernatant containing PGE_2_ was measured using a mouse enzyme-linked immunosorbent assay (ELISA) kit (R&D Systems Inc., USA) to determine the exact amount of expressed PGE_2_.

### TNF-α, IL-6, and IL- 1β Production Measurements

RAW 264.7 cells were seeded in a 24-well plate at 8.0 × 10^4^ cells/well and incubated at 37°C and 5% CO_2_ for 24 h, followed by simultaneous treatment with LPS (1 μg/ml) and DB-14 exosome (4.44 × 10^8^, 8.88 × 10^8^, 1.78 × 10^9^ particles/ml) for 24 h. The cell culture was then centrifuged at 10,000 ×*g* for 3 min to obtain a precipitate-removed supernatant. The amount of proinflammatory cytokines (IL-6, IL-1β, TNF-α) was measured using a mouse TNF-α ELISA Kit (Invitrogen, USA), a mouse IL-6 ELISA Kit (BD Biosciences, USA), and a mouse IL-1β ELISA Kit (R&D Systems Inc.).

### Melanin Production Measurements

To determine the effect of DB-14 exosome on melanin synthesis in α-MSH-induced B16F10 melanoma cells, a melanin content assay was performed. B16F10 melanoma cells were seeded into a 6-well plate at 4.0 × 10^4^ cells/well and incubated in at 37°C and 5% CO_2_ for 24 h, followed by reaction for 72 h by simultaneously treating with 200 nM α-MSH and DB-14 exosome (1.78 × 10^9^, 3.55 × 10^9^, 7.10 × 10^9^ particles/ml). Thereafter, trypsin was added to separate the cells from the plate and lysis was performed for 60 min by adding radioimmunoprecipitation assay buffer (RIPA buffer; Biosesang, Republic of Korea) containing 1 mM Na_3_VO_4_, 1 mM PMSF, and 1% protease inhibitor. Subsequently, 500 μl of 1 N NaOH was added to the pellet from which the supernatant was removed by centrifugation (4°C, 13,000 ×*g*) for 30 min and heated at 90°C for 1 h. Finally, the melanin content was transferred to a 96-well plate to measure the absorbance at 405 nm.

### Tyrosinase Activity Measurements

To investigate the effect of DB-14 exosome on tyrosinase activity in α-MSH-induced B16F10 melanoma cells, B16F10 melanoma cells were seeded into a six-well plate at 4.0 × 10^4^ cells/well and preincubated at 37°C and 5%CO_2_ for 24 h, followed by reaction for 72 h by simultaneous treatment of 200 nM α-MSH and DB-14 exosome (1.78 × 10^9^, 3.55 × 10^9^, and 7.10 × 10^9^ particles/ml). Thereafter, trypsin was added to separate the cells from the plate, and lysis was performed for 60 min by treatment with RIPA buffer containing 1 mM Na_3_VO_4_, 1 mM PMSF, and 1%proteinase inhibitor. The supernatant was acquired by centrifugation at 4°C and 13,000 ×*g* for 30 min after lysis, and the protein content contained in the supernatant was quantified using a bicinchoninic acid (BCA) kit (Bio-Rad, USA). After dark reaction for 2 h by adding 80 μl of L-DOPA made of 2 mg/ml to 20 μl of protein quantified by BCA kit, absorbance was measured at 490 nm.

### Western Blot Analysis

RAW 264.7 cells were seeded in a six-well plate at 4 × 10^5^ cells/well and cultured at 37°C and 5% CO_2_ incubator for 24 h, followed by simultaneous treatment with LPS (1 μg/ml) and DB-14 exosome (4.44 × 10^8^, 8.88 × 10^8^, 1.78 × 10^9^ particles/ml) for 24 h. Similarly, B16F10 melanoma cells were seeded in a six-well plate at 4 × 10^4^ cells/well and previously cultured at 37°C and 5% CO_2_ for 48 h, and then were simultaneously treated with 200 nM α-MSH and DB-14 exosome (1.78 × 10^9^, 3.55 × 10^9^, 7.10 × 10^9^ particles/ml) and cultured for 24 h. Thereafter, lysis was performed for 60 min by adding RIPA buffer, which contained 1 mM Na_3_VO_4_, 1 mM PMSF, and 1% proteinase inhibitor. The supernatant was then obtained by centrifugation at 4°C and 13,000 ×*g* for 30 min. The protein content of the separated supernatant was quantified using a BCA kit (Bio-Rad), and 20 μg of the quantified protein was electrophoresed using a 10% sodium dodecyl-sulfate polyacrylamide gel. After 60 min, the proteins were transferred to polyvinylidene difluoride membranes (Millipore, USA), placed in 5% skim milk dissolved in 1X TBST (Tris-buffered saline, 0.1% Tween 20), and blocked at room temperature for 2 h. The membrane was then washed three times at 10-min intervals using TBST, and the primary antibody response was performed at 4°C for 18 h using the following: iNOS antibody (1:1,000, Bio-Rad), COX-2 antibody (1:1,000, Rockland Immunochemistry, Inc., USA), TRP-1 antibody (1:5,000, Santa Cruz, USA), TRP-2 antibody (1:500, Santa Cruz), Tyrosinase antibody (1:500, Santa Cruz), MITF antibody (1:500, Cell Signaling Technology, USA), β-actin antibody clone AC-74 (1:5,000 Sigma-Aldrich), phospho (p)-p44/42 MAPK (Erk1/2)(Thr202/Tyr204) antibody (1:500, Cell Signaling technology), p-p38 MAPK antibody (1:500, Cell Signaling technology), p-Akt antibody (1:500, Cell Signaling technology), p-glycogen synthase kinase(GSK)-3β (Ser9)(5B3) antibody (1:500, Cell Signaling technology), p44/ 42 MAPK (Erk1/2) antibody (1:500, Cell Signaling technology), p38 MAPK (1:500, Cell Signaling technology), Akt (1:500, Cell Signaling technology), GSK-3β (27C10) (1:500, Cell Signaling technology). After the completion of the reaction, the membrane was washed three times with Tris-buffered saline with 0.1% Tween 20 detergent (TBST). The secondary antibodies, anti-mouse immunoglobulin (Ig)G horseradish peroxidase and anti-rabbit IgG (Jackson ImmunoResearch, USA), were diluted at a ratio of 1:10,000 and incubated at 25°C for 2 h. After 2 h, the membrane was washed thrice with TBST and then reacted with an ECL kit (Bio-Rad) to develop an imaging sensor (model GS-700, Bio-Rad). The expression of the developed proteins was quantified using the ImageJ software (NIH, USA) and plotted as a graph.

### Statistical Analysis

All experiments were repeated three times, and the results were expressed as the mean value ± standard deviation. Statistical analyses were performed via multiple comparison tests, such as Student's *t*-test and analysis of variance, to verify the significance (*p < 0.05; **p < 0.01; ***p < 0.001) of each treatment interval.

## Results

### Characterization of *L. mesenteroides* DB-14-Derived Exosome

**Fig. 1:**
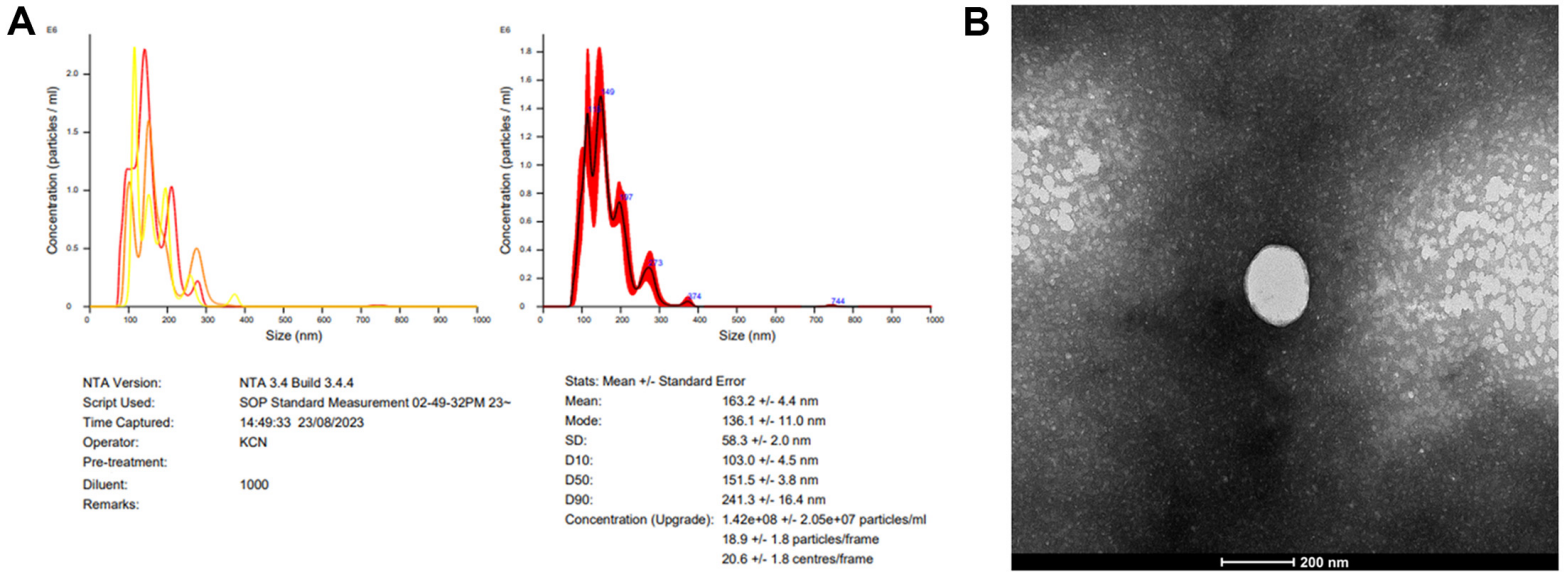
Exosome characterization of *Leuconostoc mesenteroides*-derived exosomes (DB-14 exosomes). (**A**) Nanoparticle Tracking Analysis of DB-14 exosomes. (**B**) Transmission Electron Microscopy image of DB-14 exosomes.

In previous studies, the average particle size of exosomes derived from lactic acid bacteria was reported to be 50–200 nm [32, 33]. NTA was performed in order to confirm the particle size and concentration of purified DB-14 Exosomes. The concentration of the 1000-fold diluted DB-14 exosome sample was 1.42 × 10^8^ particles/ml, with the exosomes having an average diameter of 163.2 ± 4.4 nm. Most particles were distributed between 50 and 200 nm in size (Fig. 1A). In addition, we conducted transmission electron microscopy (TEM) analysis to confirm the shape and size of DB-14 exosome, and as a result, we were able to acquire TEM image of DB-14 exosomes (Fig. 1B). These results suggest that the purified DB-14 Exosomes were of high purity; thus, further experiments were conducted using purified exosomes.

### Cell Viability

**Fig. 2:**
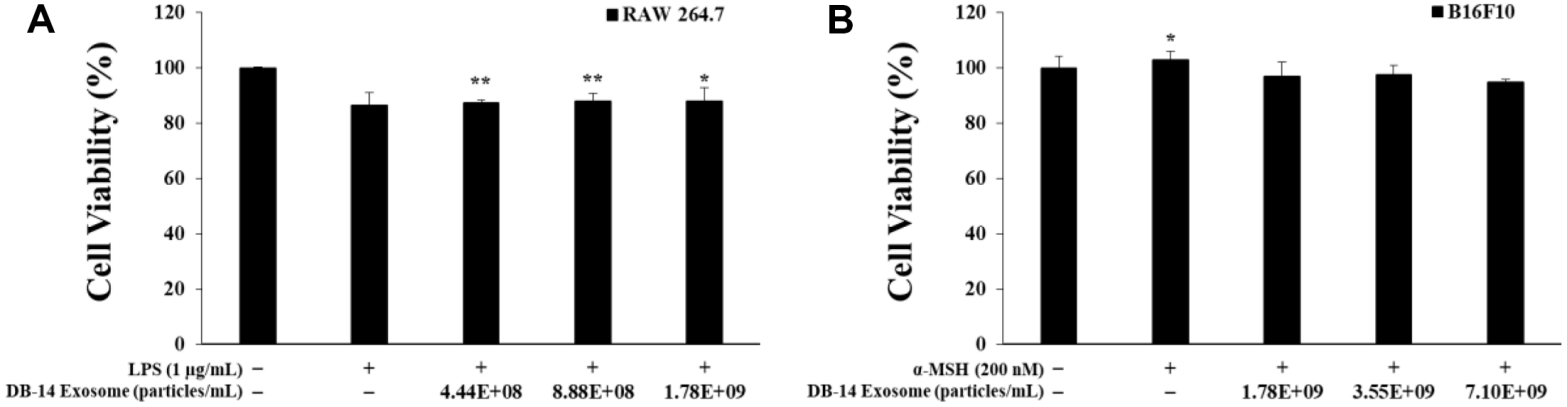
Cell viability of DB-14 exosomes on lipopolysaccharide (LPS)-stimulated RAW 264.7 cells and alpha melanocyte stimulating hormone (α-MSH)-induced B16F10 melanoma cells. The cytotoxicity of RAW 264.7 cells was determined using the 3-(4,5-dimethylthiazol-2-yl)-2,5-diphenyl-2H-tetrazolium bromide (MTT) assay for 1 μg/ml LPSstimulated cells in the presence of DB-14 exosomes (4.44 × 10^8^, 8.88 × 10^8^, 1.78 × 10^9^ particles/ml). The cytotoxicity in B16F10 cells was determined using the MTT assay for α-MSH (200 nM)-induced cells in the presence of DB-14 exosomes (1.78 × 10^9^, 3.55 × 10^9^, 7.10 × 10^9^ particles/ml). Results are expressed as percentages compared with the respective values obtained for the control. *p < 0.05; **p < 0.01.

The MTT assay is based on the ability of mitochondria to reduce MTT tetrazolium, a yellow water-soluble substrate, to 3-(4,5-dimethylthiazol-2-yl)-2,5-diphenyl-tetrazolium bromide by dehydrogenase action. To investigate the effect of DB-14 exosomes on cell survival, RAW 264.7 cells and B16F10 melanoma cells were simultaneously treated with LPS, α-MSH (200 nM), and DB-14 exosomes (4.44 × 10^8^, 8.88 × 10^8^, 1.78 × 10^9^, 3.55 × 10^9^, and 7.10 × 10^9^ particles/ml), followed by the MTT assay. Cell survival rates of 80% or more were confirmed in all of the treatment groups (Fig. 2), suggesting that DB-14 exosomes are not toxic to RAW 264.7 and B16F10 cells. Further experiments were conducted to confirm the anti-inflammatory and lightening activity of the DB-14 exosomes.

### Inhibition of NO Expression

**Fig. 3:**
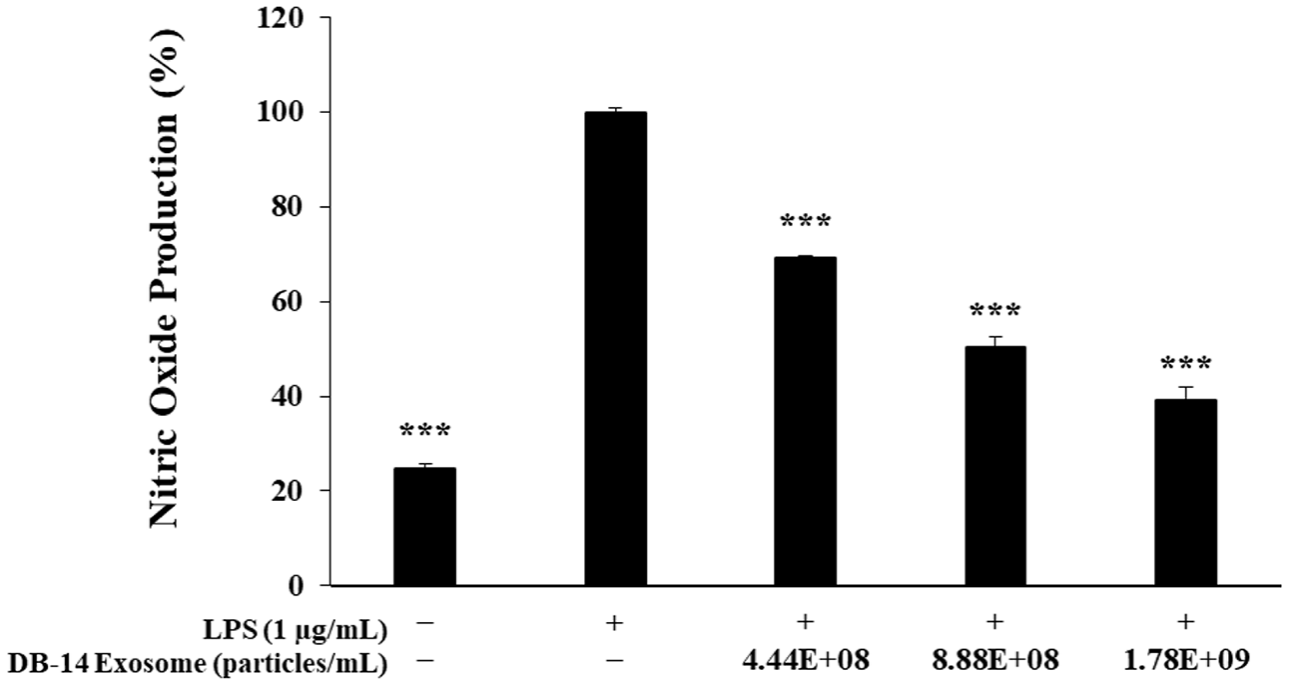
Nitric oxide production inhibition by DB-14 exosomes in LPS-stimulated RAW 264.7 cells. The production of nitric oxide in 1 μg/ml LPS-stimulated RAW 264.7 cells in the presence of DB-14 exosomes (4.44 × 10^8^, 8.88 × 10^8^, 1.78 × 10^9^ particles/ml). The results are expressed as percentages compared with the respective values obtained for the control. *p < 0.05; **p < 0.01; ***p < 0.001.

Various inflammatory mediators are expressed when inflammation is induced in the human body, and NO synthesized from iNOS is a representative inflammatory factor that causes various inflammatory diseases [34]. Therefore, in this study, to determine the effect of DB-14 exosome on NO production in the inflammatory response, RAW 264.7 cells were simultaneously treated with LPS (1 μg/ml) and DB-14 exosome (4.44 × 10^8^, 8.88 × 10^8^, and 1.78 × 10^9^ particles/ml). LPS-induced NO production was reduced in a DB-14 exosome concentration-dependent manner in RAW 264.7 cells. At the highest concentration (1.78 × 10^9^ particles/ml), DB-14 exosome reduced NO production by 60.3%, confirming that NO production was inhibited at a level similar to that of the LPS-untreated group (Fig. 3). These results suggest that DB-14 exosomes perform their anti-inflammatory activity by inhibiting the production of NO, a proinflammatory substance, in RAW 264.7 cells. Further experiments were conducted to confirm the inhibitory effects of DB-14 exosomes on the expression of various proinflammatory factors to determine their anti-inflammatory efficacy.

### Inhibition of PGE_2_ Expression

**Fig. 4:**
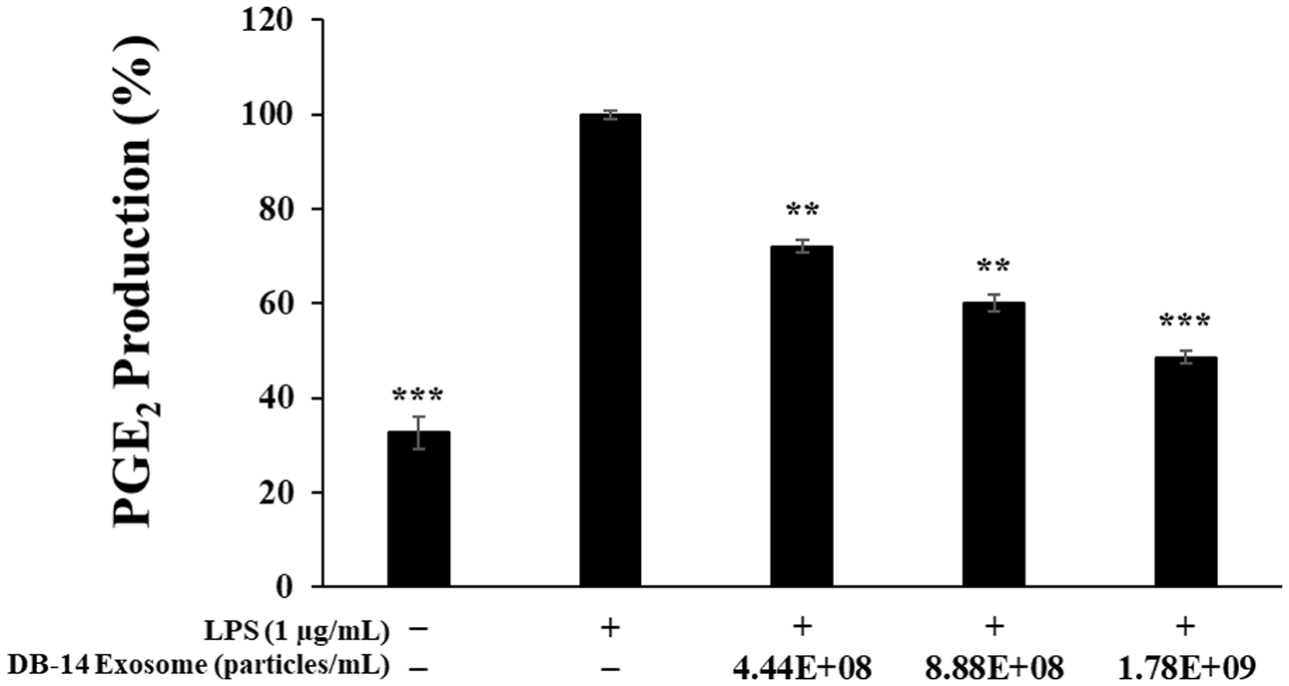
Inhibitory effects of DB-14 exosomes on prostaglandin E2 (PGE_2_) production in LPS-stimulated RAW 264.7 cells. PGE_2_ production in 1 μg/ml LPS-stimulated RAW 264.7 cells in the presence of DB-14 exosomes (4.44 × 10^8^, 8.88 × 10^8^, 1.78 × 10^9^ particles/ml). Results are expressed as percentages compared with the respective values obtained for the control. *p < 0.05; **p < 0.01; ***p < 0.001.

To evaluate the PGE_2_ production inhibitory effects of DB-14 exosome in LPS-stimulated RAW 264.7 cells, the cells were simultaneously treated with LPS (1 μg/ml) and DB-14 exosome (4.44 × 10^8^, 8.88 × 10^8^, and 1.78 × 10^9^ particles/ml), and PGE_2_ production was measured. DB-14 exosomes reduced PGE2 production in a concentration-dependent manner and exhibited high PGE_2_ inhibitory activity by inhibiting the production of PGE_2_ at the highest concentration of 1.78 × 10^9^ particles/ml (Fig. 4).

### Inhibition of Proinflammatory Cytokine Expression

**Fig. 5:**
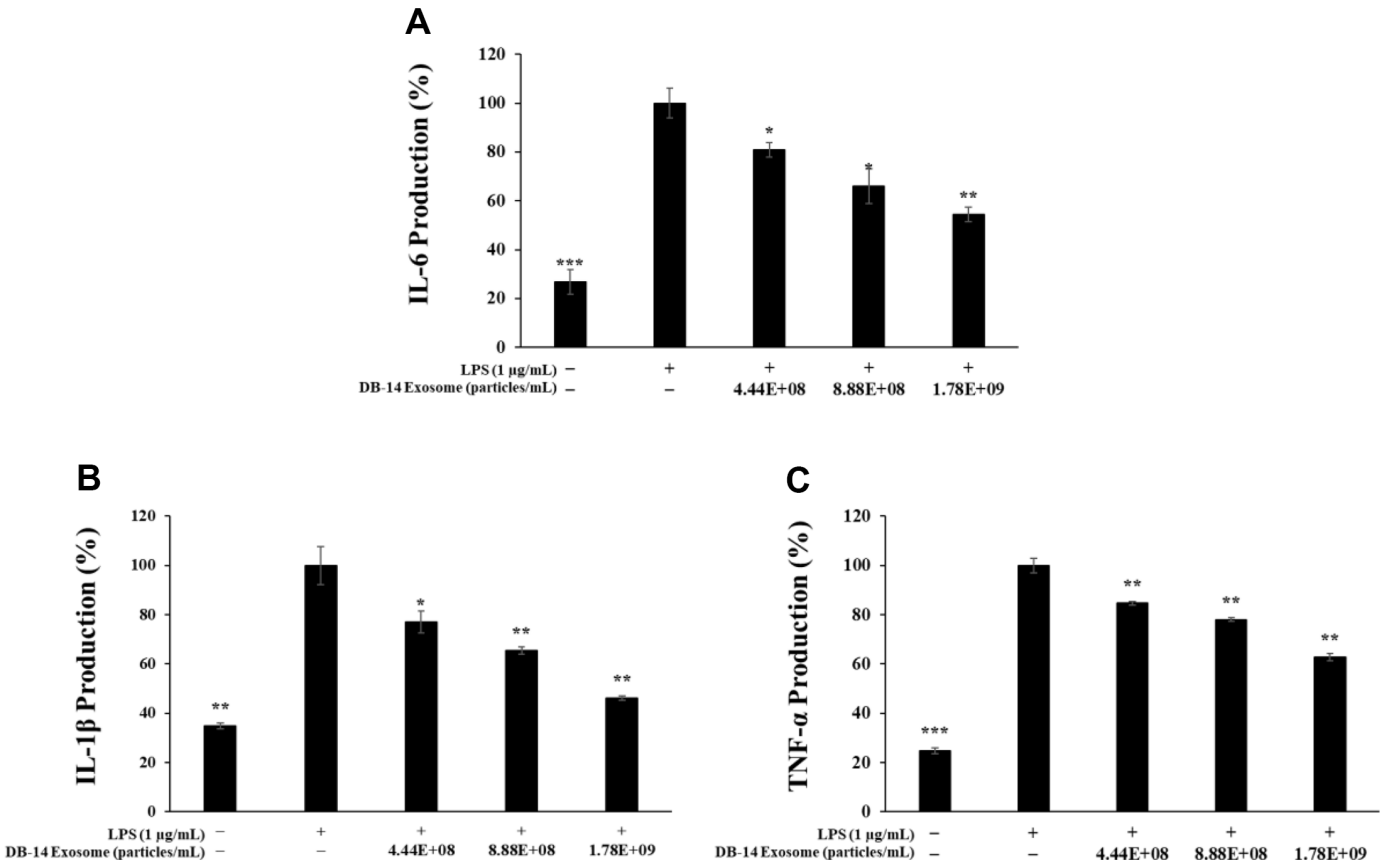
Inhibition of proinflammatory cytokines by DB-14 exosomes in LPS-stimulated RAW 264.7 cells. Production of (**A**) interleukin (IL)-6, (**B**) IL-1β, and (**C**) tumor necrosis factor alpha (TNF-α) in LPS (1 μg/ml)-stimulated RAW 264.7 cells in the presence of DB-14 exosomes (4.44 × 10^8^, 8.88 × 10^8^, 1.78 × 10^9^ particles/ml). Results are expressed as percentages compared with the respective values obtained for the control. *p < 0.05; **p < 0.01; ***p < 0.001.

Proinflammatory cytokines, such as interleukin and TNF-α, promote inflammation in the immune system and cause cellular aging and disease by upregulating the inflammatory response [35]. Therefore, in this study, the proinflammatory cytokines, TNF-α, IL-1β, and IL-6, were initially expressed in LPS-induced RAW 264.7 cells to investigate the effect of DB-14 exosomes on the reduction of inflammatory involvement factors. DB-14 exosomes significantly inhibited the LPS-induced production of IL-6, IL-1β, and TNF-α (Fig. 5). In addition, at the highest concentration of 1.78 × 10^9^ particles/ml, DB-14 exosomes inhibited IL-6, IL-1β, and TNF-α by 44.7%, 53.9%, and 37.2%, respectively. Notably, at the highest concentration of 200 μg/ml, IL-1β was inhibited at a level similar to that of the LPS-free group.

### Inhibition of Melanin Production

**Fig. 6:**
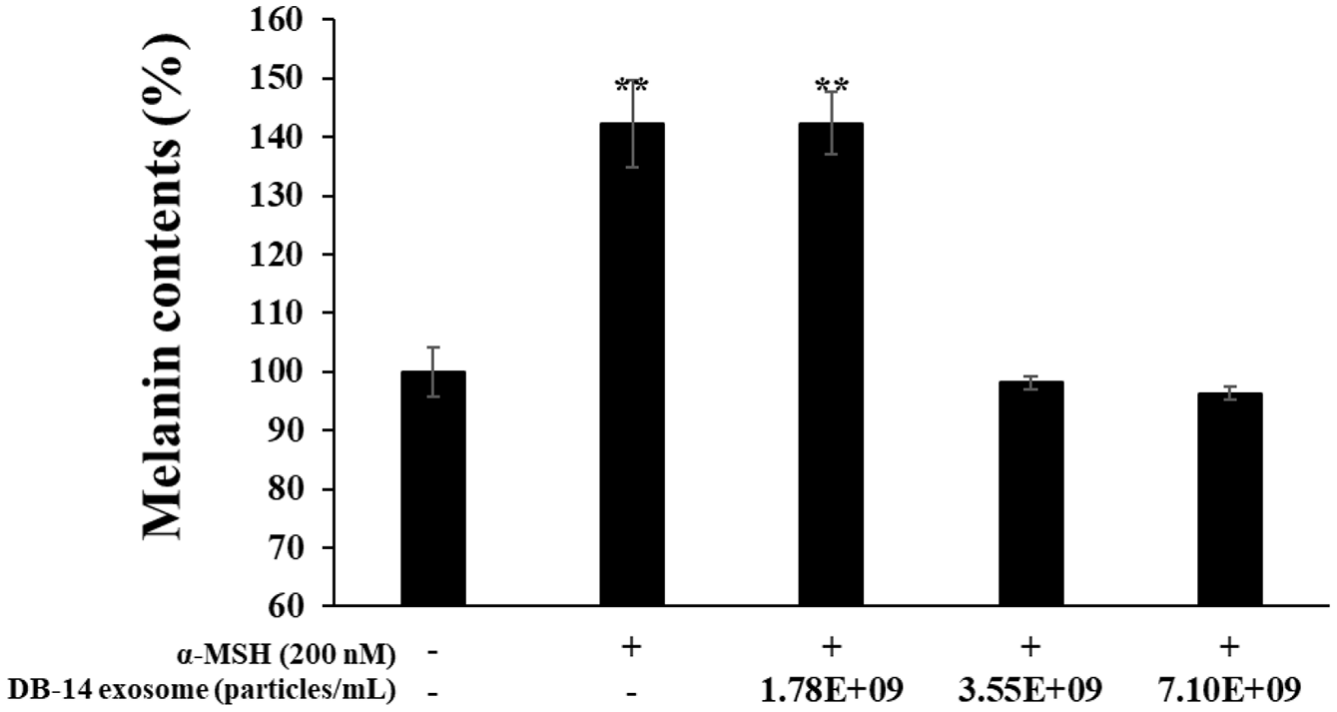
Effect of DB-14 exosomes on melanin synthesis in B16F10 melanoma cells. The production of melanin was assayed in the cell pellets of 200 nM α-MSH-stimulated cells for 72 h in the presence of DB-14 exosomes (1.78 × 10^9^, 3.55 × 10^9^, 7.10 × 10^9^ particles/ml). Data represent the means ± standard deviation (SD) with three separate experiments. *p < 0.05; **p < 0.01.

To investigate the inhibition of melanin production by DB-14 exosome in α-MSH stimulated-B16F10 melanoma cells, the cells were simultaneously treated and cultured with α-MSH (200 nM) and DB-14 exosome (1.78 × 10^9^, 3.55 × 10^9^, 7.10 × 10^9^ particles/ml), and melanin levels were measured. DB-14 exosome inhibited melanin production in a concentration-dependent manner. At the highest concentration of 7.10 × 10^9^ particles/ml, DB-14 exosome had inhibitory activity similar to that of the α-MSH-untreated group (Fig. 6). Thereafter, to determine whether DB-14 exosomes are plausible melanogenesis-reducing agents, the inhibitory activity of tyrosinase, which acts as an important rate-limiting enzyme in early-stage melanin synthesis, was evaluated.

### Inhibition of Tyrosinase Activity

**Fig. 7:**
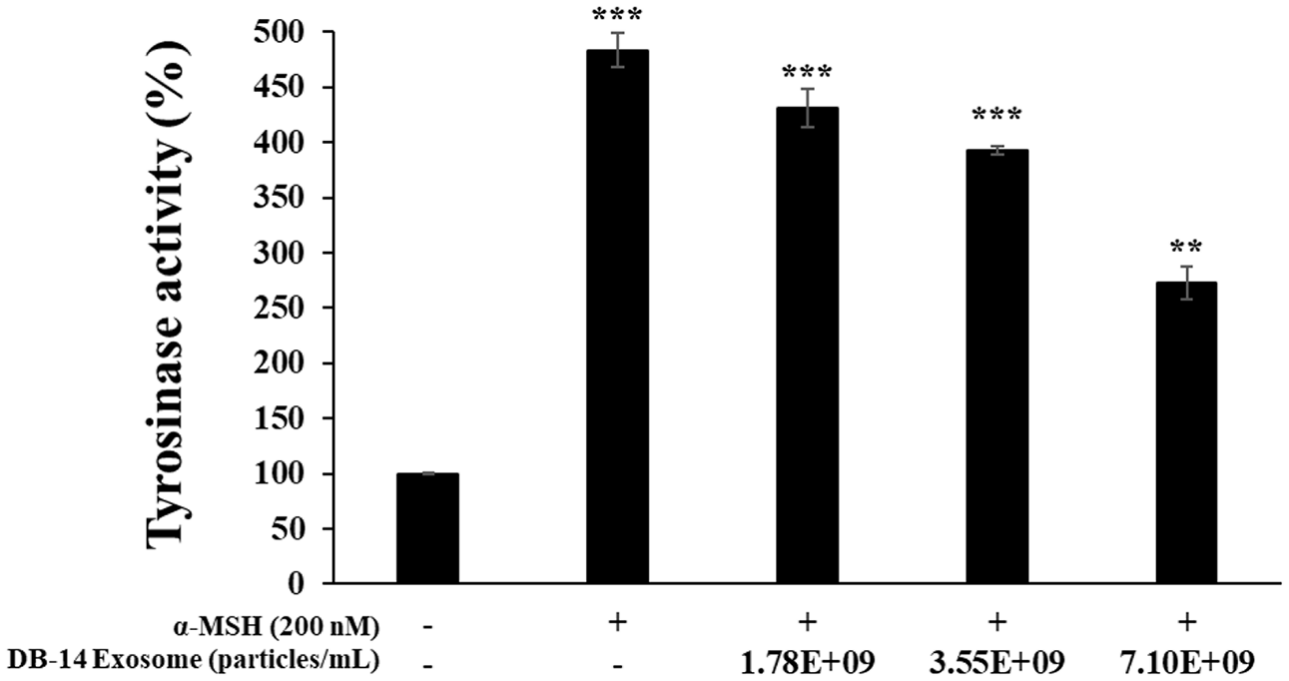
Effect of DB-14 exosomes on tyrosinase activity in B16F10 melanoma cells. The cells were stimulated with 200 nM α-MSH for 72 h in the presence of DB-14 exosomes (1.78 × 10^9^, 3.55 × 10^9^, 7.10 × 10^9^ particles/ml). The effect of DB-14 exosomes on tyrosinase activity was determined by measuring the absorbance at 490 nm. The results are expressed as a percentage of the control. Data represent the means ± standard deviation (SD) with three independent experiments. *p < 0.05; **p < 0.01; ***p < 0.001.

Tyrosinase is a key enzyme involved in melanin synthesis. The enzyme oxidizes tyrosine to dopaquinone (DOPA), and ultimately contributes to the synthesis of melanin. Therefore, inhibition of tyrosinase activity would be a direct cause of reduced melanin synthesis [36]. To investigate the effect of DB-14 exosome on tyrosinase activity, B16F10 melanoma cells were treated with α-MSH (200 nM) and DB-14 exosome (1.78 × 10^9^, 3.55 × 10^9^, 7.10 × 10^9^ particles/ml) and cultured. Intracellular proteins were then extracted to measure tyrosinase activity. In the DB-14 exosome-treated group, tyrosinase activity decreased in a concentration-dependent manner. In particular, at the highest DB-14 exosome concentration (7.10 × 10^9^ particles/ml), tyrosinase inhibitory activity was similar to that of the α-MSH untreated group (Fig. 7). Based on their inhibition of melanin synthesis and tyrosinase activity, DB-14 exosomes were judged to have significant lightening activity. Additional studies were conducted on the expression of melanin synthesis factors.

### Western Blot Analysis

**Fig. 8:**
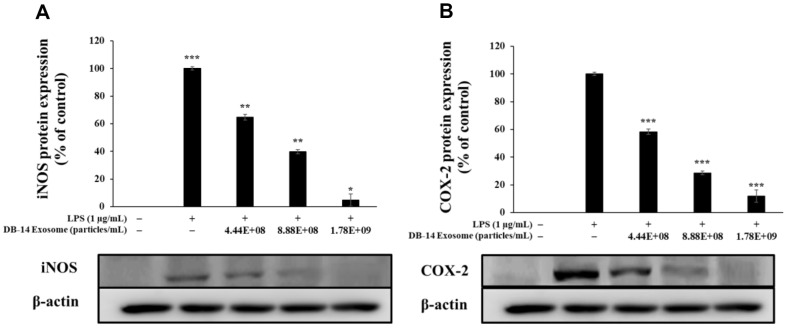
Inhibitory effects of DB-14 exosomes on inducible nitric oxide synthase (iNOS) and cyclooxygenase 2 (COX-2) protein expression in LPS-stimulated RAW 264.7 cells. Inhibitory effect of DB-14 exosome on the protein levels of (**A**) iNOS and (**B**) COX-2 in RAW 264.7 cells stimulated with LPS (1 μg/ml) in the presence of DB-14 exosomes (4.44 × 10^8^, 8.88 × 10^8^, 1.78 × 10^9^ particles/ml). Expression of iNOS, COX-2, and β-actin were determined by western blotting. Data represent the means ± standard deviation (SD) with three independent experiments. *p < 0.05; **p < 0.01; ***p < 0.001.

**Fig. 9:**
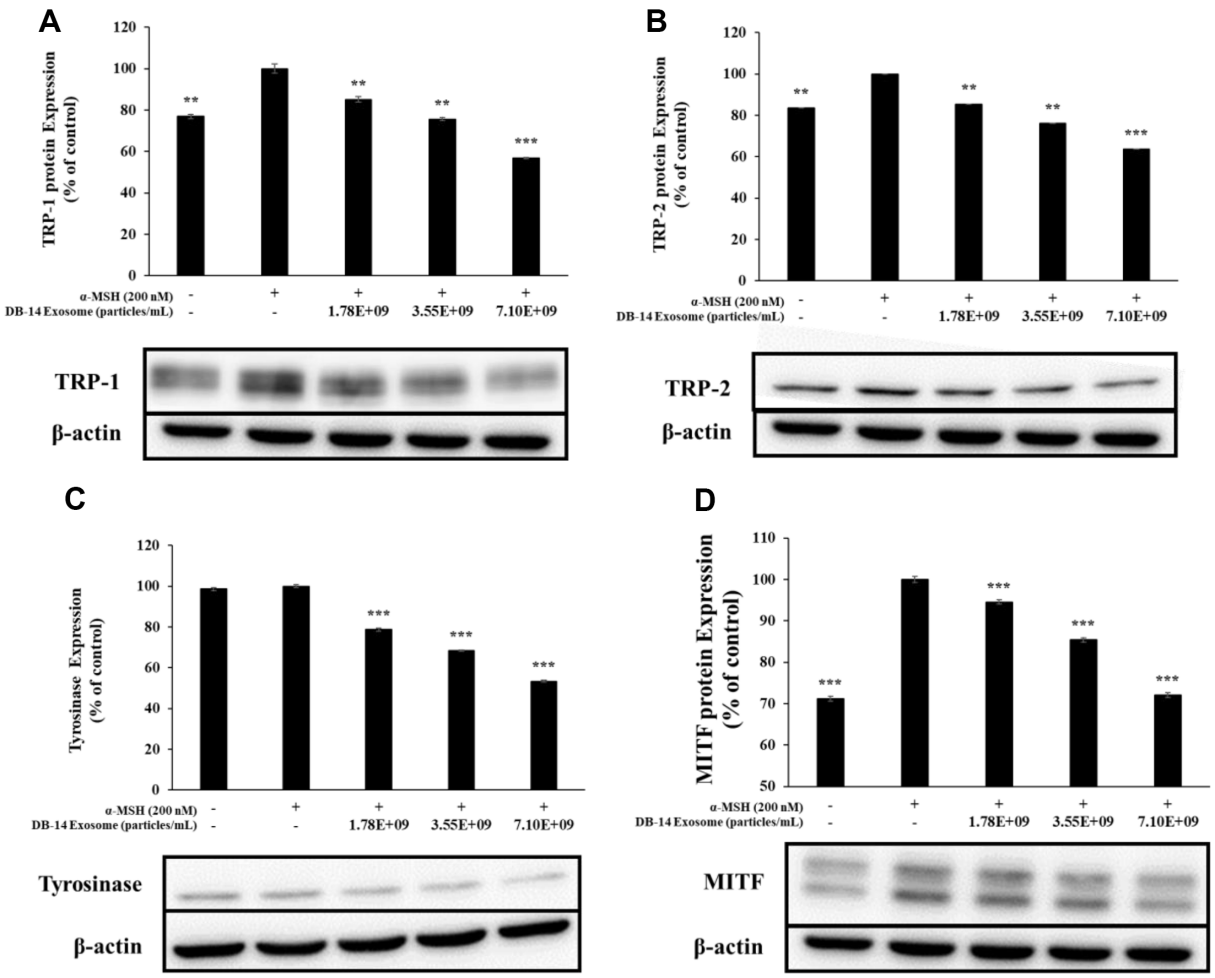
Western blot analysis of Tyrosinase related protein (TRP)-1, TRP-2, Tyrosinase and microphthalmiaassociated transcription factor (MITF) in α-MSH-induced B16F10 melanoma cells. Cells were preincubated for 48 h, and then treated with α-MSH (200 nM) and DB-14 exosomess (1.78 × 10^9^, 3.55 × 10^9^, 7.10 × 10^9^ particles/ml). (**A**) Tyrosinase related protein (TRP)-1, (**B**) TRP-2, (**C**) Tyrosinase, (**D**) MITF protein levels were analyzed using western blotting. β-actin was used as the control. Data represent the means ± standard deviation (SD) with three independent experiments. *p < 0.05; **p < 0.01; ***p < 0.001.

**Fig. 10:**
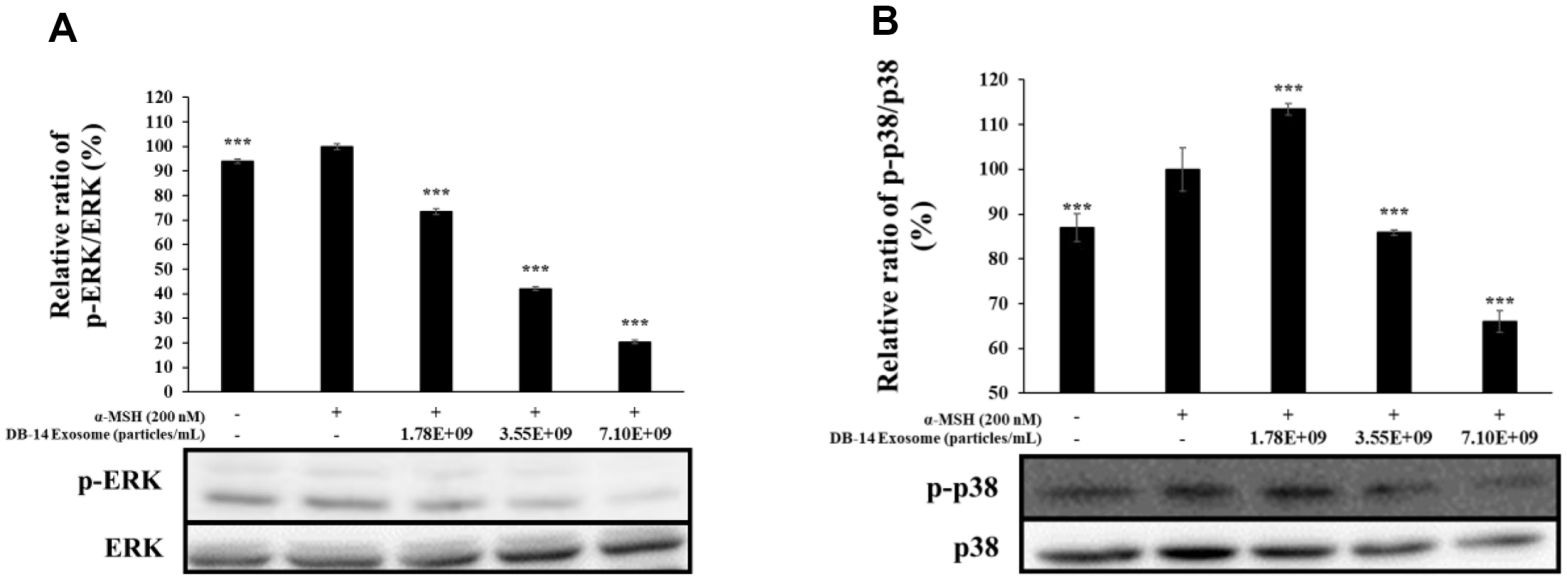
Effect of DB-14 exosomes on MAPK phosphorylation in B16F10 melanoma cells. Cells were preincubated for 48 h, and then treated with α-MSH (200 nM) and DB-14 exosomes (1.78 × 10^9^, 3.55 × 10^9^, 7.10 × 10^9^ particles/ml). (**A**) analysis for phospho (p)-ERK/ERK, (**B**) p-p38/p38. Protein levels were analyzed using western blotting. Data represent the means ± standard deviation (SD) with three independent experiments. *p < 0.05; **p < 0.01; ***p < 0.001.

**Fig. 11:**
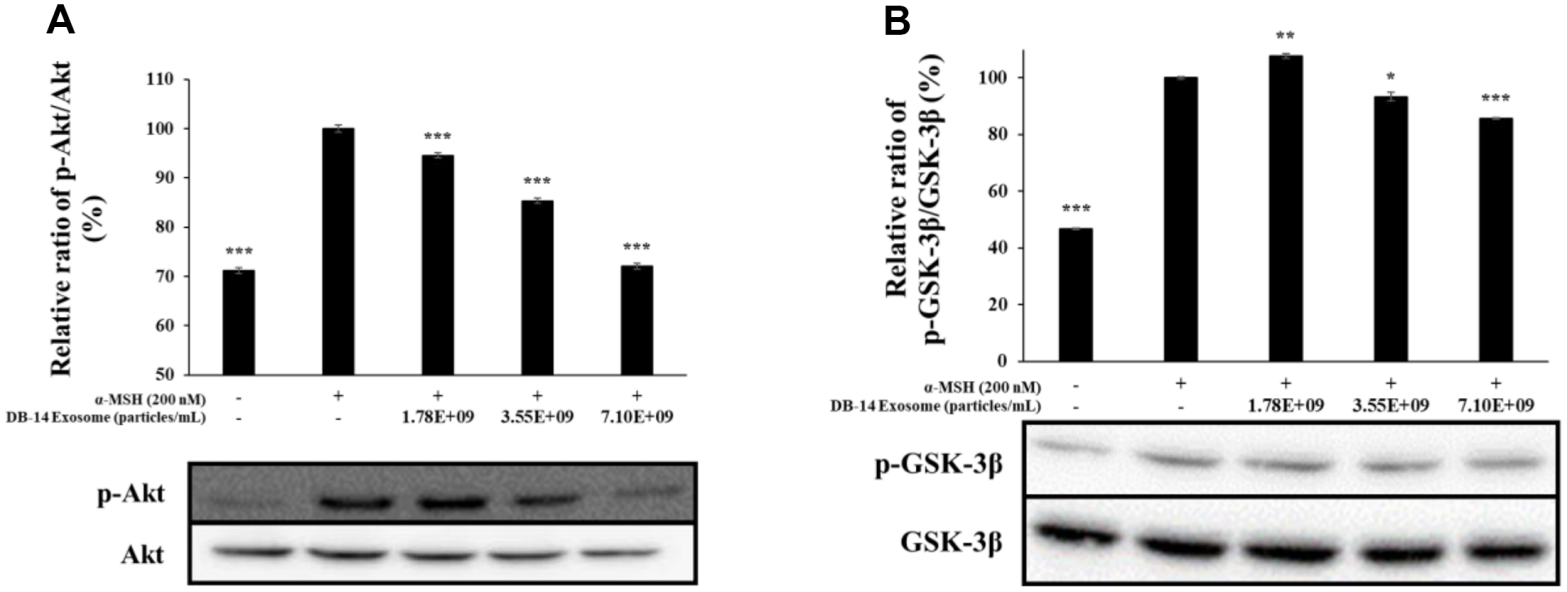
Effect of DB-14 exosomes on Akt phosphorylation in B16F10 melanoma cells. Cells were preincubated for 48 h, and then treated with α-MSH (200 nM) and DB-14 exosomes (1.78 × 10^9^, 3.55 × 10^9^, 7.10 × 10^9^ particles/ml). (**A**) Analysis for p-Akt/Akt, (**B**) p-glycogen synthase kinase (GSK)-3β/GSK-3β. Protein levels were analyzed using western blotting. Data represent the means ± standard deviation (SD) with three independent experiments. *p < 0.05; **p < 0.01; ***p < 0.001.

In macrophages, inflammatory stimuli, such as LPS, produce various inflammation-mediating factors by activating transcription factors that mediate the inflammatory response. COX-2 and iNOS stimulate the production of NO, PGE_2_, and various other inflammatory mediators to activate the inflammatory response [37, 38]. During melanogenesis, L-tyrosine is oxidized to L-DOPA by tyrosinase, oxidized again to DOPA-quinone, and DOPA-quinone is synthesized under the influence of TRP-1 and TRP-2, respectively. Moreover, microphthalmia-associated transcription factor (MITF) is intricately involved in these processes, where it synthesizes melanin. Therefore, in this study, western blotting was performed to evaluate the anti-inflammatory activity and also the inhibition of melanin synthesis of DB-14 exosomes by examining the expression of iNOS and COX-2 proteins. Furthermore, this study evaluates their lightening activity by examining the expression of TRP-1, TRP-2, tyrosinase, MITF proteins. DB-14 exosomes effectively inhibited the expression of iNOS and COX-2 (Fig. 8), a protein involved in inflammation, as well as TRP-1, TRP-2, tyrosinase, MITF proteins, which are involved in the melanogenesis reaction (Fig. 9). In addition, the phosphorylation levels of the MAPK and Akt pathways were investigated to confirm the mechanism of melanin synthesis inhibition. As a result, it was confirmed that DB-14 exosomes inhibited the phosphorylation of the ERK, p38 (Fig. 10) such and Akt, GSK-3β (Fig. 11), and it was confirmed that melanin synthesis was finally inhibited by inhibiting the phosphorylation of the aforementioned pathway.

Thus, DB-14 exosomes can be used as anti-inflammatory and lightening drugs to inhibit the proteins involved in inflammatory reactions and melanin synthesis.

## Discussion

In this study, anti-inflammatory and whitening efficacy experiments were conducted using exosomes derived from *L. mesenteroides* DB-14, a native Korean flower symbiotic lactic acid bacterium isolated from the NIBR. The exosomes were successfully isolated using the TFF system, and their size, concentration, and morphology were confirmed by NTA and TEM.

DB-14 exosomes effectively inhibited the activity of nitric oxide at concentrations (4.44 × 10^8^, 8.88 × 10^8^, and 1.78 × 10^9^ particles/ml) which did not affect the growth of RAW 264.7 macrophages. In addition, these exosomes effectively suppressed the activity of pro-inflammatory cytokines such as TNF-α, IL-6, IL-1β, and prostaglandin E2, and similarly, western blot experiments confirmed that it effectively suppressed the activity of iNOS and COX-2, known as major factors related to inflammation.

To evaluate the skin-whitening activity of DB-14 exosomes, we found that melanin synthesis and tyrosinase activity were significantly reduced at nontoxic concentrations of the DB-14 exosomes (1.78 × 10^9^, 3.55 × 10^9^, 7.10 × 10^9^ particles/ml). A western blot experiment was conducted to confirm the effect of DB-14 exosomes on the expression level of TRP-1, TPR-2, Tyrosinase, and MITF, which are known to be factors involved in melanin formation, and as a result, it was confirmed that DB-14 exosomes inhibit the expression of these factors in a concentration-dependent manner. MITF is the most important factor among them, stimulated by a number of factors and involved in melanin synthesis. The phosphorylation levels of MAPK and Akt pathways were measured to determine the mechanism by which MITF is inhibited. As a result, it was confirmed that DB-14 exosomes effectively inhibited the phosphorylation of ERK, and p38, and likewise inhibited the phosphorylation of Akt and its sub-mechanism, GSK-3β. These results suggest that DB-14 exosomes inhibit the phosphorylation of the MAPK and Akt pathways, thereby inhibiting the expression of MITF and ultimately inhibiting melanin synthesis.

This study suggests that the exosomes derived from *L. mesenteroides* DB-14, a native Korean *C. japonica* flower symbiotic lactic acid bacterium have excellent anti-inflammatory activity and at the same time have a strong melanin synthesis inhibitory ability. Therefore, DB-14 exosomes have shown potential for use as strong inflammatory treatments and skin-whitening agents, and to utilize DB-14 exosomes as a functional agents, a clicical research on their functional efficacy in human applications, as well as constituent proteins and nucleic acids of DB-14 exosomes should be conducted.
